# Early cessation of exclusive breastfeeding and associated factors in Ethiopia: a systematic review and meta-analysis

**DOI:** 10.3389/fnut.2025.1500077

**Published:** 2025-04-25

**Authors:** Tadele Emagneneh, Chalie Mulugeta, Abebaw Alamrew, Betelhem Ejigu, Wagaw Abebe

**Affiliations:** ^1^Department of Midwifery, Woldia University, Woldia, Ethiopia; ^2^Department of Medical Laboratory Science, Woldia University, Woldia, Ethiopia

**Keywords:** breastfeeding, determinants, early cessation, exclusive breastfeeding, Ethiopia

## Abstract

**Background:**

Early cessation of exclusive breastfeeding poses significant risks to infant health by depriving them of essential immune-boosting and nutritional benefits found in breast milk. The First 1,000 Days, from conception to a child’s second birthday, is a critical window for growth and development. EBF during this period plays a vital role in ensuring optimal nutrition, strengthening immunity, and reducing childhood morbidity and mortality. However, early discontinuation of EBF remains a major public health concern in Ethiopia, contributing to malnutrition and increased susceptibility to infections.

**Aim:**

Despite global efforts to promote exclusive breastfeeding, early cessation remains a growing concern. This systematic review and meta-analysis assess the prevalence and associated factors of early cessation of EBF to provide evidence-based insights for improving breastfeeding practices.

**Methods:**

We conducted a comprehensive search of published papers in Scopus, PubMed, Google Scholar, and Web of Science databases, up to March 1, 2024, without any restrictions on publication date. Records were screened, data extracted, and the risk of bias was independently assessed by five reviewers. Study heterogeneity was evaluated using Cochrane I^2^ statistics, while publication bias was assessed through the Egger-Begg test and funnel plots. A random-effects model was used to estimate the pooled prevalence of early cessation of EBF.

**Results:**

The analysis included 3,726 participants from 9 studies: five cross-sectional, two case–control, and two cohort studies. The overall pooled prevalence of early cessation of EBF was 43.31% (95% CI: 27.30–59.32). Several factors were significantly associated with early cessation, including urban residence (OR = 2.01, 95% CI: 1.7–2.30), lack of maternal education (OR = 2.79, 95% CI: 1.86–3.72), maternal employment (OR = 2.88, 95% CI: 1.36–4.41), poor knowledge of breastfeeding (OR = 2.08, 95% CI: 1.45–2.70), delayed initiation of breastfeeding (OR = 2.88, 95% CI: 1.57–2.18), and being a first-time mother (OR = 2.08, 95% CI: 1.45–2.70).

**Conclusion:**

The pooled prevalence of early cessation of EBF in Ethiopia was found to be 43.31% (95% CI: 27.30–59.32), indicating that nearly half of Ethiopian infants are not breastfed exclusively for the recommended 6 months. Factors significantly associated with early cessation include urban residence, non-educated mom, employment, poor maternal knowledge, delayed breastfeeding initiation, and Primipara (being a first-time mother). These findings underscore the need for targeted interventions, such as community-based education programs, workplace breastfeeding support policies, and strategies to promote early breastfeeding initiation and improve maternal knowledge. Addressing these factors could help reduce early cessation, increase sustained EBF rates, and improve child health outcomes.

## Introduction

Breast milk is considered the most natural and complete source of nutrition for newborns and infants, offering safe and sufficient nourishment (1). In addition to providing essential nutrients like proteins, fats, vitamins, and minerals, breast milk supports immune function, protecting against conditions such as pneumonia, gastrointestinal infections, urinary tract infections, and other illnesses (2). The World Health Organization (WHO) recommends exclusive breastfeeding (EBF) for the first 6 months, without any additional liquids, including water (3). Beyond this period, breastfeeding should continue for up to 2 years, supplemented with suitable solid foods (4).

The first 1,000 days of a child’s life, from conception to age 2 years, are crucial for growth, development, and overall health (5). During this period, children have increased nutritional needs, and inadequate care can lead to lasting negative effects, including higher rates of morbidity and mortality (6). Notably, the early cessation of EBF contributes to half of all child deaths globally, often resulting from wasting, stunting, and micronutrient deficiencies (7). Moreover, the rising prevalence of childhood overweight and obesity is associated with early EBF cessation, increasing the lifetime risk of severe health issues and accounting for 7.1% of adult deaths (8).

Research indicates that introducing complementary foods before 6 months should be carefully evaluated, considering the benefits of exclusive breastfeeding, the risks of formula feeding, and the quality of supplementary foods (1, 2). Early supplementation has been linked to short-term issues like slow growth and increased infections, as well as long-term risks such as atopic conditions, diabetes, obesity, and impaired neuromuscular development (5, 9).

Early cessation of EBF within the first 6 months significantly impacts a child’s nutrition and long-term well-being, primarily due to the loss of breast milk’s immune-boosting benefits, which support healthy growth and reduce the risk of illnesses (5). Studies link early cessation to higher rates of diarrhea, fever, acute respiratory infections (ARIs), underweight, and wasting (10, 11).

EBF provides over 50% protection against these adverse outcomes, and sustaining it for 6 months could prevent approximately 10% of related illnesses, particularly diarrhea, fever, and ARIs (11, 12). The earlier EBF cessation occurs, the higher the risk, with diarrhea, stunting, wasting, and underweight affecting 50% of children who stop EBF early (11, 13). Notably, the risk of diarrhea progressively increases the earlier EBF is ceased, with the highest risks observed when cessation occurs at 0–2 months, followed by 2–4 months, and 4–6 months (12, 14, 15). Longer EBF durations are associated with reduced rates of these and other long-term health problems, highlighting its critical role in preventing ARIs, undernutrition, and promoting lifelong health (14, 16).

Early cessation of exclusive breastfeeding (EBF) remains a significant public health concern in Ethiopia, with reported rates ranging from 21.5 to 75% (15, 17–21). These variations reflect disparities in maternal education, healthcare access, economic conditions, and cultural norms, despite national efforts such as breastfeeding promotion programs and maternity leave policies. Understanding these differences is essential for addressing gaps in EBF practices and improving maternal and child health outcomes.

Regional and socioeconomic variations in EBF cessation directly impact infant nutrition, increasing the risk of malnutrition and infections. Identifying the key factors driving these disparities can help policymakers develop targeted, region-specific strategies to promote sustained EBF. This systematic review and meta-analysis provide a comprehensive estimate of early EBF cessation in Ethiopia and its determinants, offering evidence-based insights to guide interventions aimed at reducing early cessation rates and supporting optimal breastfeeding practices.

## Methods

### Study settings

This systematic review and meta-analysis focused on studies conducted in Ethiopia, a country in the Horn of Africa with a diverse population and varying regional health challenges. The review aimed to include data from both urban and rural settings, reflecting the wide-ranging socio-economic conditions and healthcare access across different regions. Studies were selected from various sources, including hospitals, health centers, and community-based studies to ensure a comprehensive representation of breastfeeding practices nationwide. The studies included in this review were conducted across diverse settings in Ethiopia, spanning both resource-limited rural areas and urban regions. However, the inclusion criteria were based on the study population rather than the specific study setup, ensuring that the findings represent Ethiopia’s diverse socio-economic and healthcare contexts without bias toward particular study environments. This approach allowed us to capture a broad range of factors influencing early cessation of exclusive breastfeeding.

### Study reporting

**Figure 1:**
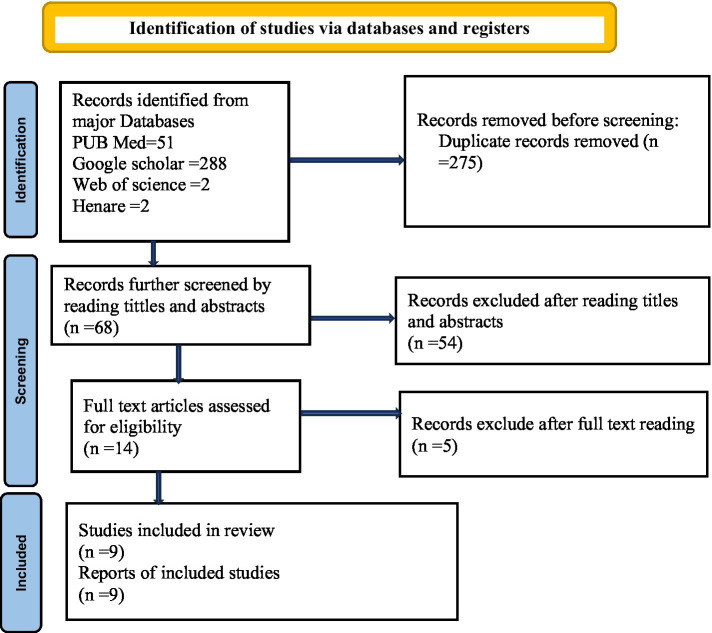
PRISMA flowchart illustrating the selection process for studies included in the systematic review and meta-analysis on the early cessation of exclusive breastfeeding and its associated factors in Ethiopia, 2024.

This systematic review and meta-analysis aimed to evaluate the prevalence of early cessation of EBF and its associated factors in Ethiopia. The study was conducted following the PRISMA 2020 guidelines for systematic reviews and meta-analyses (22), with the study selection process depicted in Figure 1 as a PRISMA flow diagram (Figure 1). The protocol of this systematic review was registered in the International Prospective Register of Systematic Reviews (PROSPERO) (protocol ID: CRD42024488979).

### Selection criteria

#### Inclusion criteria

The studies considered in this review met the following criteria: (1) Studies conducted in Ethiopia involving mothers with children aged 0–24 months, encompassing both rural and urban populations across all regions, and focusing on specifically breastfeeding practices. (2) Mothers of having healthy infants up to 6 months of age, (3) were written in English, (4) All observational studies focusing on early cessation of EBF and its associated factors are included without publication year restriction.

### Exclusion criteria

Studies were excluded if they met any of the following criteria: (1) included infants identified as severely ill during the first 6 months of life, (2) failed to report outcomes associated with the early cessation of exclusive breastfeeding (EBF), (3) lacked sufficient data, such as odds ratios and confidence intervals, or (4) were systematic reviews or meta-analyses.

### Search strategies

This systematic review and meta-analysis aimed to assess the prevalence of early cessation of EBF and its associated factors in Ethiopia. In this study, researchers surveyed four international databases—Medline/PubMed, Scopus, Web of Science, and Hinari—along with Google Scholar, to identify articles published until March 1, 2024. A comprehensive search strategy was employed, with no study design or publication date restrictions. The search utilized a combination of keywords, MeSH terms, Boolean operators such as “OR” and “AND”, and snowball searching to identify relevant studies (Supplementary file 1). The keywords used for searching the international databases included AND (Breastfeeding OR Breast Feeding OR (Exclusive AND Breastfeeding (All Fields)) OR Lactation OR Infant Nutrition OR Infant Feeding) and (Problems OR Barriers OR Difficulties OR Determinants) OR Early Discontinuation OR Early Cessation OR Early Termination (Facilitate OR Promote OR Support OR Motivate) OR Duration OR Optimal OR Maintenance OR Guideline Adherence (All fields)) AND (Ethiopia OR all regions OR city administrations (All Fields) OR 9 regions OR two city administrations (All Fields) OR Ethiopia) OR Amhara OR Tigray OR Oromia OR Somali OR Afar OR Benishangul OR Gambelia OR Harari OR SNNP OR Addis-Ababa OR Dir Diwa) AND (English) AND (Article)))). The review followed the PRISMA 2020 guidelines for systematic reviews and meta-analyses, with the study selection process depicted in Figure 1 as a PRISMA flow diagram.

### Data extraction

After removing duplicates using Mendeley referencing software, five reviewers independently extracted data from the full-text articles selected for the study, utilizing a modified Johanna Briggs Institute (JBI) data abstraction format (23). Discrepancies among reviewers were resolved through discussion and consensus, ensuring a high level of agreement (Cohen’s kappa coefficient > 0.60). The extracted data included the author’s name, publication year, study location (region), sample size, prevalence of early cessation of EBF with 95% confidence intervals (CI), and factors linked to the early cessation of EBF.

### Measurement of outcomes

The meta-analysis focused on two main objectives: (i) estimating the pooled prevalence of early cessation of exclusive breastfeeding (EBF) in Ethiopia, expressed as a percentage, and (ii) identifying factors associated with early cessation of EBF, analyzed using adjusted odds ratios (AOR) from primary studies. The study aimed to provide a comprehensive synthesis of evidence to inform strategies for sustaining EBF practices and reducing its early cessation in Ethiopia by modifying contributing factors. Only studies that defined early cessation of EBF as the introduction of supplemental foods or a complete shift to alternative nutrition before 6 months in children aged 2 years or younger were included in this systematic review and meta-analysis (19–21, 24).

### Quality evaluation

Five authors (TE, AA, BE, WA, and CM) independently assessed the quality of all included studies using a modified Newcastle-Ottawa Scale (NOS) (25), a widely recognized tool for evaluating the quality of observational studies in systematic reviews and meta-analyses as all the retained studies are observational; which comprised, two case–control studies, two cohort studies, and five cross-sectional studies. The quality assessment process rigorously examined key domains, including methodological rigor, comparability of study groups, accuracy of outcome measurements, and appropriateness of statistical analyses. Discrepancies in scoring among the five authors were thoroughly discussed and resolved through consensus to ensure objectivity and consistency. Studies that achieved a score of seven or higher out of a possible 10 were classified as high-quality and deemed suitable for inclusion in the review (26) (Supplementary file 2).

### Data synthesis and statistical analysis

Data from the included articles were extracted into Microsoft Excel and analyzed using STATA version 17. A meta-analysis was performed to estimate the overall prevalence of early cessation of EBF using a random-effects model (27). Heterogeneity among the studies was assessed with Cochrane’s Q test and I^2^ statistics (28, 29), revealing significant heterogeneity (I^2^ = 98.6%, *p* < 0.001). Due to the high heterogeneity (I^2^ > 50%), the Der Simonian and Laird random-effects model was applied for the analysis (29).

Publication bias was evaluated using Egger’s and Begg’s regression tests at a significance level of 0.05 (30, 31), and no evidence of publication bias was found (Egger’s test: *p* = 0.089). The pooled prevalence, along with its 95% confidence intervals (CI), was displayed in a forest plot. Similarly, factors associated with early cessation of exclusive breastfeeding (EBF) were analyzed using pooled log odds ratios with 95% CI and presented in a separate forest plot.

## Results

A total of 343 articles were retrieved from various databases, and 68 unique studies remained after removing duplicates. During the initial title and abstract screening, 18 articles were excluded for being irrelevant. A further 36 articles were excluded after abstract review for not meeting the inclusion criteria. Subsequently, 14 full-text articles were assessed against the eligibility criteria, resulting in the exclusion of 5 studies for not reporting the outcome of interest. Ultimately, 9 studies met the criteria and were included in the final meta-analysis (Figure 1).

### Characteristics of included studies

Among the 9 articles published in Ethiopia up to March 1, 2024, a total of 3,726 study participants were involved in determining the pooled prevalence of early cessation of exclusive breastfeeding and associated factors. The study designs included five cross-sectional studies, two case–control studies, and two cohort studies. Three of the studies were from Amhara regions (17, 19, 24). Two studies were from south nation nationalities of people of Ethiopia region(SNNP) (15, 32). The remaining four studies are from Tigray (33), Oromia (20), Afar (18) and Somali (21) regions. The quality assessment of the studies yielded scores ranging from 7 to 9 (Table 1).

**Table 1:** The study characteristics included in the systematic review and meta-analysis on prevalence of early cessation of EBF and associated factors in Ethiopia.

Authors	Regions	Study design	Sample size	Prevalence of early cessation EBF	Quality
Beyene et al. (18)	Afar	Cross-sectional	484	32	8
Kelkay et al. (19)	Amhara	cross-sectional	344	21.5	7
Getachew et al. (65)	SNNP	Case–control study	336	……	8
Adugnaw et al. (17)	Amhara	Cross-sectional	426	45	7
Ibrahim et al. (21)	Somali	Cross-sectional	292	57.3	9
Temesgen et al. (15)	SNNP	Cross-sectional	436	29.3	8
Yeneabat et al. (24)	Amhara	Case–control	592	-----	9
Kebede et al. (20)	Oromia	Cross-sectional	313	75	9
Techane et al. (33)	Tigray	Cohort	503	------	9

SNNP, South nation nationality and people.

### Prevalence of early cessation of EBF in Ethiopia

**Figure 2:**
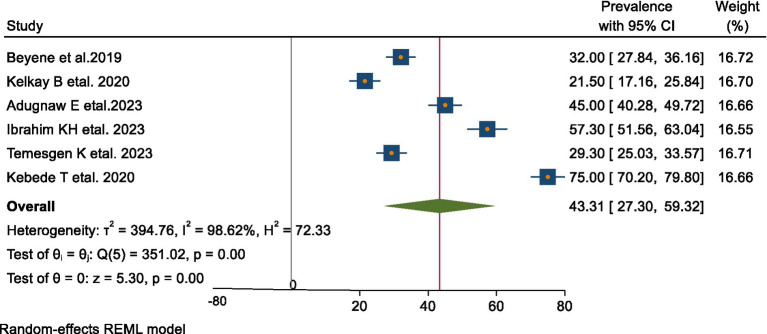
Forest plot of the pooled prevalence of early cessation of EBF in Ethiopia.

This systematic review and meta-analysis estimated the pooled prevalence of early cessation of exclusive breastfeeding in Ethiopia at 43.31% (95% CI: 27.30–59.32), as illustrated in Figure 2. The broad confidence interval reflects considerable variability among the included studies, likely due to differences in methodologies and study populations. This highlights the importance of interpreting the pooled estimate cautiously, with careful consideration of the context and characteristics of each study.

### Heterogeneity and publication bias

#### Publication bias

**Figure 3:**
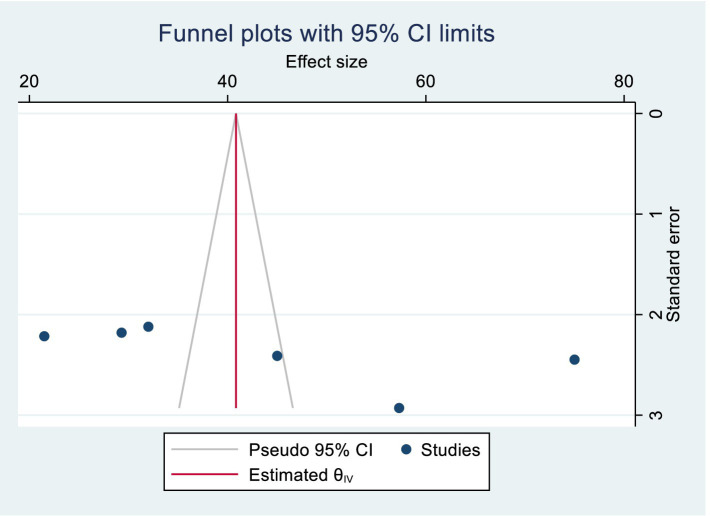
Funnel plot for included studies on the prevalence of early cessation EBF in Ethiopia.

The funnel plot showed a symmetric distribution, and Egger’s test produced a *p*-value of 0.089, indicating no evidence of publication bias in this systematic review and meta-analysis (Figures 2, 3). Consequently, a trim-and-fill analysis was not required.

#### Subgroup analysis

Significant heterogeneity was observed among the included studies, with an I^2^ value of 98.62%. To investigate potential sources of heterogeneity, a subgroup analysis was conducted based on study design, response rate, and publication year. The analysis revealed that the highest prevalence of early cessation of exclusive breastfeeding was reported in community-based cross-sectional studies, at 43.78% (95% CI: 27.91–59.65, I^2^ = 96.86%, *p* < 0.001). In contrast, the lowest prevalence was found in institution-based cross-sectional studies, at 42.82% (95% CI: 10.76–74.88, I^2^ = 99.37%, *p* = 0.01).

Regarding response rates, studies with a 100% response rate reported the highest prevalence, at 60% (95% CI: 30.60–89.40, I^2^ = 98.69%, *p* < 0.001), while studies with less than a 100% response rate showed a lower prevalence of 34.94% (95% CI: 19.86–50.02, I^2^ = 97.72%, *p* < 0.001).

Subgrou*p* analysis by publication year indicated similar prevalence rates across different periods, with the highest prevalence observed in studies published after 2020, at 43.78% (95% CI: 27.91–59.32, I^2^ = 96.86%, *p* < 0.001). Detailed findings are provided in Table 2.

**Table 2:** Subgroup analysis of the pooled prevalence of early cessation of exclusive breastfeeding in Ethiopia.

Variables	Characteristics	Included studies	Number of study participants	Prevalence with 95% CI	I2, *p*-value
Region	Afar	1	465	32.00 [27.84, 36.16]	…, < 0.001
	Amhara	2	767	33.23 [10.20, 56.26]	98.06, 0.005
	Oromia	1	313	75.00 [70.20, 79.80]	…, < 0.001
	SNNP	1	433	29.30 [25.03, 33.57]	…, < 0.001
	Somali	1	281	57.30 [51.56, 63.04]	…, < 0.001
Study design	Institution based cross-sectional	3	1,119	42.82 [10.76, 74.88]	99.37, 0.009
	Community-based cross-sectional	3	1,140	43.78 [27.91, 59.65]	96.86, 0.000
Response rate	100%	2	1,578	60.00 [30.60, 89.40]	98.69, 0.000
	<100%	4	2,083	34.94 [19.86, 50.02]	97.72, 0.000
Publication year	after 2020	3	1,979	43.78 [27.91, 59.65]	96.86, 0.000
	2020 and before	3	1,682	42.82 [10.76, 74.88]	99.37, 0.009
Overall		9	3,661	43.31 [27.30, 59.32]	98.62, <0.001

### Sensitivity analysis

A leave-one-out sensitivity analysis was performed to investigate potential sources of heterogeneity in the pooled prevalence of early cessation of EBF in Ethiopia. The analysis demonstrated that the findings were robust and not disproportionately influenced by any single study. When each study was excluded individually, the pooled prevalence ranged from 36.93% (95% CI: 24.60–49.27%) to 47.68% (95% CI: 31.09–64.27%), confirming the stability of the results (see Table 3).

**Table 3:** Sensitivity analysis showing the prevalence of early cessation of exclusive breastfeeding in Ethiopia, with 95% confidence intervals, calculated by omitting each study individually.

Omitted study	Prevalence in %	95% CI	*p*-value
Beyene et al. (18)	45.59	[26.73, 64.44]	< 0.001
Kelkay et al. (19)	47.68	[31.09, 64.27]	< 0.001
Adugnaw et al. (17)	42.98	[23.39, 62.58]	< 0.001
Ibrahim et al. (21)	40.54	[22.12, 58.96]	< 0.001
Temesgen et al. (15)	46.13	[27.70, 64.56]	< 0.001
Kebede et al. (20)	36.93	[24.60, 49.27]	< 0.001

### Factors associated with early cessation of exclusive breastfeeding

Several studies highlighted various factors contributing to the early cessation of exclusive breastfeeding. After compiling and synthesizing these findings, three key themes were identified: socio-demographic factors, obstetric factors specific to the mothers, and aspects related to breastfeeding counseling and knowledge. Furthermore, the timing of breastfeeding initiation and associated challenges emerged as significant contributing factors.

### Socio-demographic factors

#### Association of urban residence with early cessation of EBF

**Figure 4:**
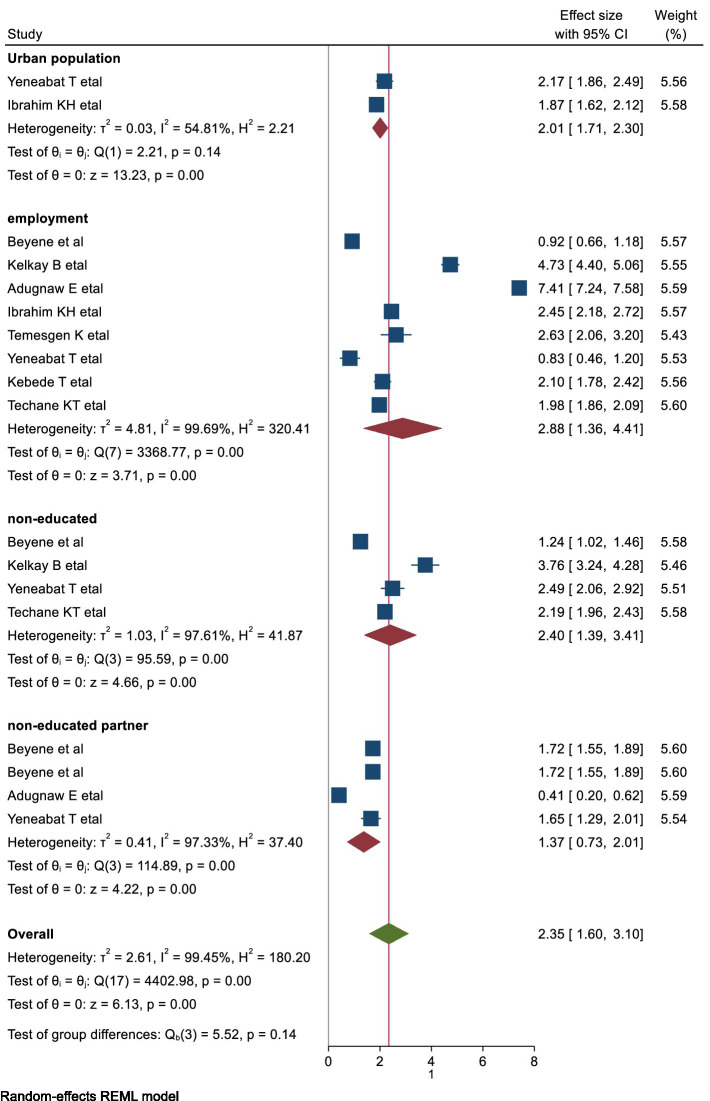
Forest plots the association of socio-demographic factors with early cessation of EBF in Ethiopia.

This meta-analysis included two studies that assessed the relationship between urban residence and early cessation of exclusive breastfeeding. The findings revealed that women living in urban areas were twice as likely to discontinue exclusive breastfeeding early compared to those in rural areas (pooled OR: 2.01, 95% CI: 1.70–2.30) (Figure 4).

#### The association of education level with early cessation EBF

In this systematic review and meta-analysis, maternal education was categorized into two groups: educated and non-educated. Four studies were included to evaluate the association between maternal education and early cessation of exclusive breastfeeding. The analysis showed that non-educated mothers were 2.4 times more likely to discontinue exclusive breastfeeding before 6 months compared to educated mothers (pooled OR: 2.4, 95% CI: 1.39–3.41) (Figure 4).

### The association of employment with early cessation of exclusive breastfeeding

Our analysis shows that employed mothers are more likely to cease exclusive breastfeeding early compared to non-employed mothers. This meta-analysis, which included eight studies, examined the relationship between maternal employment status and the early cessation of EBF. The results showed that employed mothers were near 3 times more likely to cease exclusive breastfeeding before 6 months compared to mothers who were housewives (pooled OR: 2.88, 95% CI, 1.36–4.41).

### The obstetric factors

#### The association of primipara with early cessation of exclusive breastfeeding

**Figure 5:**
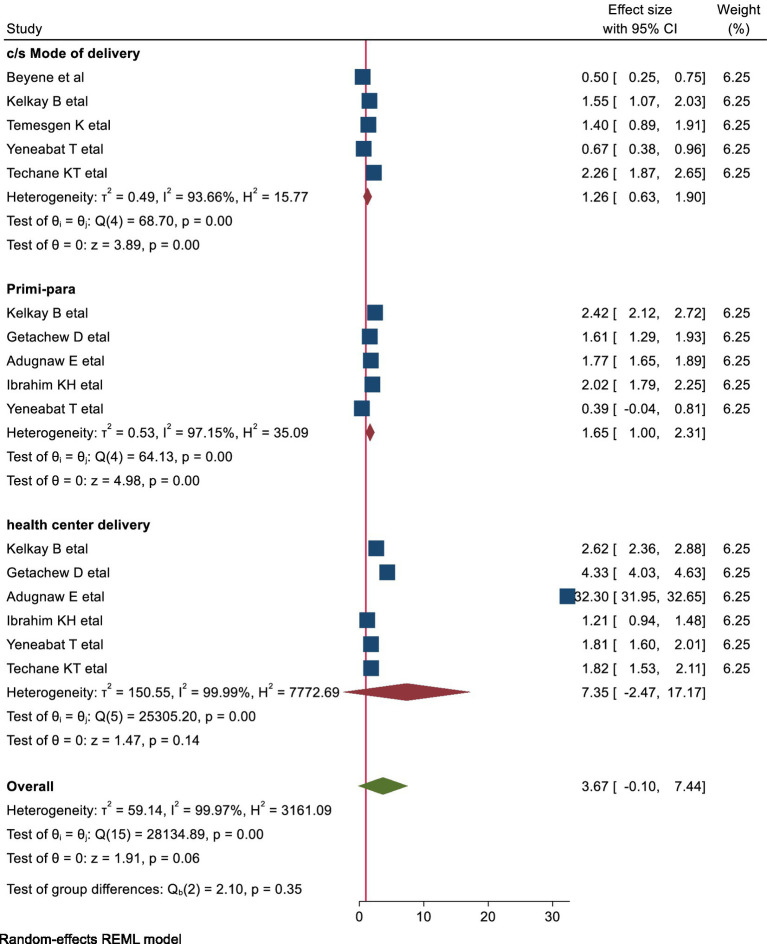
Shows the forest plots of obstetric factors with early cessation of EBF.

This meta-analysis included five studies to examine the association between primiparity and early cessation of EBF. The analysis revealed that primiparous women were 1.65 times more likely to cease exclusive breastfeeding before 6 months compared to multiparous women (pooled OR: 1.65, 95% CI: 1.00–2.30) (Figure 5).

### EBF counseling and the mother’s knowledge of EBF

#### The association of maternal knowledge with early cessation of EBF

**Figure 6:**
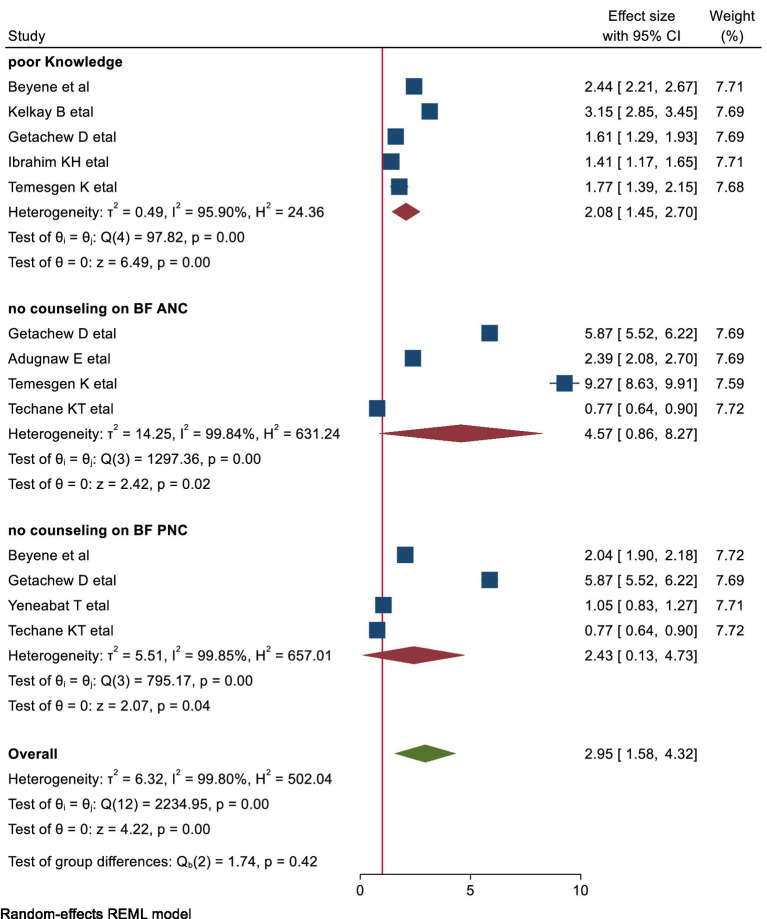
shows the forest plots of EBF counseling and knowledge with early cessation of EBF in Ethiopia.

This meta-analysis included five studies to explore the link between poor knowledge and early cessation of EBF. The results showed that women with poor knowledge about the benefits of EBF were twice as likely to stop breastfeeding before 6 months compared to those with better knowledge (pooled OR: 2.08, 95% CI: 1.45–2.70) (Figure 6).

### Late initiation of breastfeeding and related problems

#### The association of late initiation of breast-feeding with early cessation of EBF

**Figure 7:**
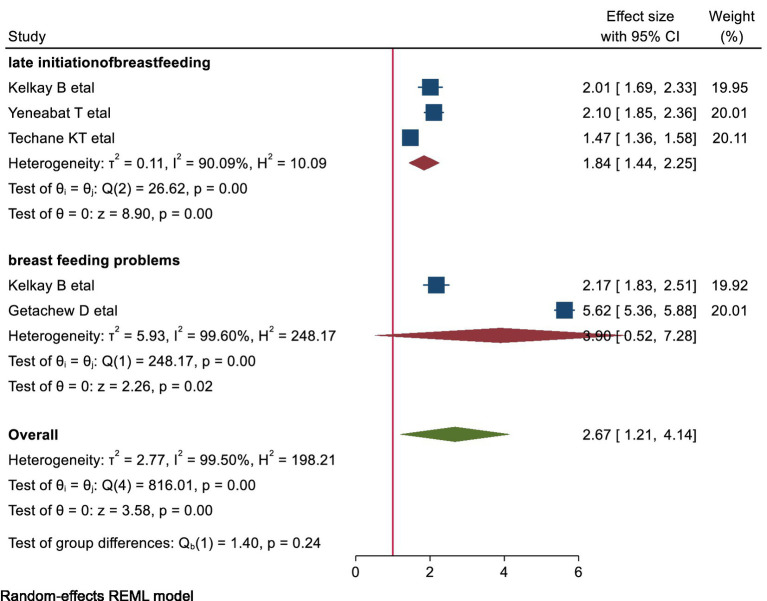
shows the association of late initiation of breastfeeding and related factors with early cessation of EBF.

This meta-analysis included three studies to examine the relationship between late initiation of breastfeeding and early cessation of EBF. The findings indicated that mothers who initiated breastfeeding late were 1.84 times more likely to cease exclusive breastfeeding before 6 months compared to those who initiated breastfeeding early (pooled OR: 1.84, 95% CI: [1.44–2.25]; Figure 7).

## Discussion

Early cessation of EBF has significant implications for both immediate and long-term health outcomes (9). EBF during infancy is essential for optimal growth, immune system development, and the prevention of infections and chronic diseases (1, 34). Evidence indicates that early cessation of EBF can result in inadequate nutrition and increased susceptibility to childhood infections such as diarrhea and pneumonia, which are leading causes of childhood morbidity and mortality (11, 35, 36). Moreover, these early-life health issues have been linked to long-term consequences. Suboptimal immune programming during infancy, due to insufficient breastfeeding, may predispose individuals to chronic conditions such as obesity, cardiovascular diseases, and type 2 diabetes later in life (9, 37). This association highlights how disruptions in early nutrition and health can contribute to adult morbidity and mortality, emphasizing the critical importance of sustaining EBF for the recommended 6 months.

This systematic review and meta-analysis provide a comprehensive estimate of the pooled prevalence of early cessation of exclusive breastfeeding (EBF) in Ethiopia and its associated factors. The findings indicate a pooled prevalence of 43.31% (95% CI: 27.30–59.32), highlighting early cessation as a persistent public health concern. Although Ethiopia has implemented various breastfeeding promotion initiatives and maternity leave policies, these efforts may not sufficiently address key challenges, particularly among urban and employed mothers. Societal pressures, limited workplace accommodations, and inadequate breastfeeding support systems remain significant barriers to sustained EBF (38).

This high prevalence underscores gaps in current breastfeeding promotion policies, necessitating targeted interventions to improve breastfeeding practices. Expanding workplace breastfeeding accommodations, reinforcing maternity leave protections, and strengthening community-based education programs are crucial strategies. Additionally, integrating breastfeeding counseling into routine antenatal and postnatal care, promoting peer support networks, and leveraging digital health tools for remote breastfeeding education could enhance maternal support and improve EBF rates. Future research should focus on region-specific challenges to tailor interventions that address disparities in EBF cessation across Ethiopia. By implementing multi-level interventions at policy, healthcare, and community levels, Ethiopia can improve exclusive breastfeeding rates and enhance long-term maternal and child health outcomes. Notably, this prevalence closely matches findings from Saudi Arabia (39). However, it exceeds the rates reported in several other countries, including China (40), Australia 27% (41), Sri Lanka 28.8% (42), India (43). Furthermore, it surpasses the estimates from systematic reviews conducted in Brazil (44) and cohort study done in Portugal (45). These variations could be attributed to differences in cultural practices, maternal education levels, healthcare support systems, and breastfeeding promotion programs. Additionally, variations in study design, sample size, and data collection methods may have influenced the reported rates, highlighted the importance of contextual and methodological considerations when compared such findings across different settings. The significant heterogeneity observed (I^2^ = 98.62%) in the included studies warrants further examination of potential contributing factors. One possible source of heterogeneity is regional differences, as the studies were conducted across various Ethiopian regions, which may have differing socio-economic conditions, healthcare systems, and cultural practices influencing breastfeeding behaviors. Additionally, methodological variations among studies, such as differences in sample size, data collection methods, and measurement of breastfeeding duration, could contribute to the variability in results. These factors underscore the need for caution when generalizing the findings and suggest that future research should aim to standardize methodologies and consider regional contexts in order to reduce heterogeneity and improve the consistency of the findings.

The analysis identified six key factors associated with early cessation of EBF: urban residence, lack of maternal education, maternal employment, poor knowledge about EBF, late initiation of breastfeeding, and nulliparity.

Urban residence was found to significantly influence early cessation of exclusive breastfeeding (EBF), with urban women being twice as likely to stop EBF before 6 months compared to their rural counterparts. This disparity can be attributed to several factors, including higher employment rates in urban areas, which limit time for breastfeeding due to workplace commitments and shorter maternity leave. Additionally, urban women may rely more on formula feeding for convenience. Urban healthcare practices and cultural norms often perceive breastfeeding as less practical, particularly in public or professional settings, further discouraging EBF (46). These factors highlight the need for targeted interventions, such as workplace support, community programs, and culturally sensitive breastfeeding promotion, to address barriers and support urban mothers in sustaining EBF in work place. Our finding aligns with studies from Kenya (47) and a mixed-method study in China (40), which similarly reported higher rates of early EBF cessation among urban residents. However, it contrasts with lower rates of early cessation reported in another study in China (48) and a study done in Canada (49). These discrepancies may be influenced by differences in urban living conditions, cultural norms, workplace policies, and access to breastfeeding support services. In urban areas, factors such as greater work-related demands, shorter maternity leave, and increased reliance on formula feeding may contribute to higher cessation rates. Conversely, in some urban settings like Canada, robust breastfeeding promotion initiatives and supportive workplace policies may help mitigate these challenges, resulting in lower cessation rates. These contextual factors highlight the need for targeted interventions to support urban mothers in sustaining EBF practices.

Maternal education is a crucial socio-demographic factor affecting the early cessation of exclusive breastfeeding (EBF). Women without formal education are more likely to stop breastfeeding before 6 months compared to those with at least primary education. This gap may be due to limited knowledge about the benefits of EBF and reduced exposure to breastfeeding promotion programs, which often target literate populations. As a result, a lack of awareness can lead to misconceptions about breastfeeding, increasing reliance on formula feeding or early introduction of complementary foods (46). Educated mothers tend to have better access to healthcare services, including lactation support and breastfeeding programs, and are more aware of the long-term health benefits of EBF for both the mother and child. This understanding increases the likelihood of engaging in breastfeeding support programs and sustaining breastfeeding for the recommended duration, ultimately improving maternal and child health outcomes (50). However, maternal empowerment is not solely defined by education; it also includes access to healthcare, financial stability, and supportive work policies. Women with greater access to maternal and child health services are more likely to receive breastfeeding counseling, which reinforces the importance of EBF. Financial stability and maternity leave policies further influence a mother’s ability to continue breastfeeding, as economic constraints may require an early return to work. Additionally, a mother’s autonomy in decision-making—free from family pressures, cultural norms, or workplace constraints—greatly impacts EBF continuation. Strengthening maternal empowerment through multi-dimensional interventions can foster an environment that supports sustained EBF and improves infant health outcomes (38). Our finding was consistent with studies conducted in Saudi Arabia (39), India (43), and Siri Linka (42), where lower education levels were similarly associated with early cessation. However, it contradicts findings from a study in Italy (51), which reported no significant association between education level and EBF practices. Furthermore, the findings of our study is higher than rates reported in systematic reviews and meta-analyses from low- and middle-income countries (52), Brazil (44), China (40), Saudi Arabia (53), and Portugal (45). This disparity may be due to Cultural norms and social influences in the study setting that may differ from those in the compared countries, where traditional practices and family advice often discourage sustained EBF, particularly among less-educated women. Additionally, disparities in healthcare accessibility and support systems may contribute to the discrepancy. Countries like Brazil, China, and Portugal, with robust maternal health programs and breastfeeding promotion initiatives, offer better support for EBF, whereas the study setting may lack these resources, disproportionately impacting women with lower education levels. Methodological differences, such as variations in study design, sample size, and data collection, may also explain the differences in rates. These factors emphasize the need for targeted interventions to address educational gaps, promote culturally sensitive breastfeeding education, and enhance healthcare support for mothers.

In our study, occupation significantly influenced early cessation of exclusive breastfeeding (EBF), with employed mothers more likely to cease EBF before 6 months compared to housewives. This aligns with previous studies, which highlight several contributing factors. Work-related stress can negatively impact mothers’ ability to maintain EBF, affecting both their physical and emotional capacity. Moreover, the lack of workplace breastfeeding support—such as private spaces for pumping and flexible break times—can discourage continued breastfeeding (46). Employment often limits the time available for breastfeeding, whereas housewives may have more flexibility to care for their infants and sustain EBF. Additionally, limited maternity leave or inadequate workplace policies that fail to support breastfeeding mothers exacerbate early cessation. These challenges underscore the need for improved workplace policies, including extended maternity leave and better breastfeeding facilities, to support working mothers and reduce early cessation rates. Addressing these issues through policy reforms and workplace support programs is essential for improving breastfeeding outcomes, ultimately benefiting both maternal and child health (54, 55). This finding is consistent with studies from Saudi Arabia (53) Kenya (47) and a systematic and meta-analysis study done in low and middle-income countries (52). However, it is notably higher than findings from studies in Canada, Australia (56), Brazil (57), China (40), India (43), and France (58). The discrepancy in findings regarding occupation and early cessation of exclusive breastfeeding (EBF) can be attributed to several factors. Firstly, differences in workplace policies, such as maternity leave duration and breastfeeding accommodations, may explain the variation. In countries like Canada, Australia, Brazil, China, India, and France, supportive policies such as longer maternity leave and workplace facilities for breastfeeding help employed women continue EBF longer. In contrast, the study setting may lack these policies, making it more difficult for employed women to sustain EBF. Secondly, cultural attitudes toward breastfeeding and employment vary across countries. In countries with lower cessation rates, such as Canada and France, breastfeeding is more socially accepted in the workplace, encouraging employed women to continue. However, in the study setting, social norms may discourage breastfeeding in public or at work. The nature of employment also plays a role. In countries with lower cessation rates, employed women may have more flexible jobs, making breastfeeding easier, whereas in the study setting, women may work in less flexible sectors. Lastly, methodological differences, such as sample size, study design, and data collection methods, could contribute to the discrepancy. These factors emphasize the need to consider local policies, cultural norms, and employment conditions when examining the relationship between occupation and early cessation of EBF.

The timing of breastfeeding initiation plays a critical role in the early cessation of exclusive breastfeeding (EBF). In this study, women who did not initiate breastfeeding within the first hour after birth were more likely to cease EBF before 6 months. This finding aligns with studies from Saudi Arabia (39), Portugal (45), and a systematic and meta-analysis study in middle and lower-income countries (52). The link between late initiation and early cessation is crucial. Late initiation often results from factors such as delivery complications, delayed skin-to-skin contact, or lack of lactation support. These challenges can hinder the establishment of breastfeeding, reduce maternal confidence, and make it more difficult to maintain EBF (59). As a result, addressing the causes of late initiation—through improved healthcare practices, timely skin-to-skin contact, and better lactation support—is essential. Such interventions will promote sustained breastfeeding, enhance maternal confidence, and ultimately reduce early cessation rates However, this finding is higher than the results from China (40), which may be due to differences in healthcare practices, cultural norms, and breastfeeding support. In China, breastfeeding initiation is often more strongly supported by healthcare providers and family members, reducing the impact of delayed initiation. Local factors such as healthcare infrastructure and maternal education may also influence the relationship between early initiation and EBF duration. These variations emphasize the need for region-specific interventions to promote early breastfeeding initiation and sustain EBF.

In Ethiopia, limited knowledge about the benefits of exclusive breastfeeding (EBF) significantly contributes to its early cessation. Our findings indicate that women with insufficient knowledge of EBF’s advantages are twice as likely to stop breastfeeding before 6 months compared to those who are more informed. Many mothers may not fully understand how EBF prevents infant illnesses and supports optimal growth. However, the quality of this knowledge plays a crucial role (46). Mothers who acquire breastfeeding knowledge from reliable sources—such as formal education, healthcare support, or media campaigns—are better equipped to continue breastfeeding for the recommended duration (60). Healthcare professionals and breastfeeding support programs offer evidence-based guidance, while media campaigns help raise awareness and shape positive community attitudes toward breastfeeding. In contrast, lack of access to quality information or reliance on misinformation can lead to early cessation (61). This highlights the need for targeted educational interventions and awareness campaigns to ensure mothers receive accurate, comprehensive breastfeeding education from various trusted sources, which could help promote sustained breastfeeding practices and reduce early cessation rates. This finding is consistent with studies from Siri Linka (42), Kenya (47), and Congo (62), but lower than results from Saudi Arabia (63), where similar gaps in knowledge contributed to early cessation. However, this study is higher than that reported in studies from China (40), a systematic and meta-analysis study done in low and middle-income countries (52) and Canada (49), which may benefit from more established breastfeeding education, public health campaigns, and healthcare infrastructure. The difference may be attributed to these countries having more established breastfeeding education programs, widespread public health campaigns, and stronger healthcare infrastructure to support and promote. Further methodological differences, such as sample size, study design, and data collection methods, could contribute to the discrepancy. These disparities highlight the need for increased educational efforts and better access to breastfeeding support to improve the rates of exclusive breastfeeding in Ethiopia.

In our study, primiparous mothers (first-time mothers) were found to be 1.7 times more likely to stop exclusive breastfeeding before 6 months compared to multiparous mothers. This is likely due to a combination of factors, including a lack of breastfeeding experience, which can decrease confidence and increase the likelihood of difficulties such as nipple pain, low milk supply, or poor latch. These challenges can lead to frustration and early discontinuation of breastfeeding (60). First-time mothers may also face unrealistic expectations, social pressures, and insufficient support from family, peers, or healthcare providers, which can further exacerbate the situation. Inadequate counseling and guidance from healthcare systems also contribute to these challenges. Without prior experience, first-time mothers may feel less confident in their ability to successfully breastfeed, which can ultimately contribute to early cessation (60). This highlights the importance of providing enhanced support and counseling to first-time mothers to help them navigate the challenges of breastfeeding, ensuring better breastfeeding outcomes and longer durations of exclusive breastfeeding. Similar findings have been reported in studies from Saudi Arabia (53). However, it is higher than the studies from Brazil (64), and France (58). These differences may stem from countries like Brazil and France, which have stronger healthcare support systems, including breastfeeding education, helping primiparous women breastfeed longer. In contrast, countries like Ethiopia with less comprehensive support may experience higher cessation rates.

In general, Early cessation of EBF remains a significant public health concern in Ethiopia, with a high prevalence driven by factors such as urban residence, maternal employment, low education, poor knowledge, late breastfeeding initiation, and primiparity. Despite existing policies, challenges like workplace constraints and inadequate breastfeeding support persist. Addressing these barriers through workplace accommodations, community education, and improved healthcare support can help sustain EBF and improve maternal and child health outcomes. Region-specific interventions and policy enhancements are crucial to reducing early cessation and promoting optimal breastfeeding practices.

## Limitations

The studies included in this review were mostly from a few specific regions and had small sample sizes in some areas. This limits the ability to apply the findings to the entire population. Additionally, although most Ethiopian researchers conduct their work in English, restricting this review to English-only publications may introduce language bias by excluding valuable research in local or regional languages. While English is the nationally endorsed language for academic and research purposes, promoting broader accessibility, this limitation could compromise the review’s comprehensiveness and affect the generalizability of its findings. Despite these limitations, the rigorous meta-analytic approach and inclusion of studies from diverse settings enhance the reliability of the findings. Future research should employ longitudinal designs, standardized data collection methods, and more representative population samples to strengthen the evidence base.

## Conclusion

The high prevalence of early cessation of EBF in Ethiopia (43.31%) remains a significant public health concern. Key contributing factors include urban residence, maternal employment, lower maternal education, delayed breastfeeding initiation, and primiparity. Urban mothers, in particular, face additional barriers such as work-related pressures, formula feeding, and changing lifestyles, while rural mothers often benefit from stronger cultural support and fewer employment-related constraints. Addressing this challenge requires targeted, multi-level interventions to promote sustained breastfeeding practices. Strengthening breastfeeding education, expanding maternal support services, and enhancing community awareness programs are essential strategies. Policymakers should integrate evidence-based breastfeeding support into national health policies, including workplace accommodations and maternal education programs. Healthcare providers must ensure consistent breastfeeding counseling and postpartum follow-up, while community organizations play a crucial role in fostering supportive environments through peer support networks and awareness campaigns.

Future research should focus on region-specific challenges and assess the effectiveness of these interventions across diverse settings. Strengthening breastfeeding support systems at the policy, healthcare, and community levels is vital for improving maternal and child health outcomes in Ethiopia.

## Data Availability

The data analyzed in this study is subject to the following licenses/restrictions: the datasets used and analyzed during the current study are available from the corresponding author (Tadele Emagneneh) on reasonable request. Requests to access these datasets should be directed to tadeleemagneneh@gmail.com.
