# Supplementary material for: Early cessation of exclusive breastfeeding and associated factors in Ethiopia: a systematic review and meta-analysis
Source: Front Nutr. 2025 Apr 25;12:1500077. doi: 10.3389/fnut.2025.1500077 (PMC12089787; doi:10.3389/fnut.2025.1500077)
Supplement: Supplementary file 1 [file Table_1.docx]

**Supplemental Table 1: Literature search strategy for the present systematic literature review on prevalence and barriers to cessation of exclusive breast-feeding in Ethiopia**.

| 1.((Breastfeeding OR Breast Feeding OR (Exclusive AND Breastfeeding (All Fields)) OR Lactation OR Infant Nutrition OR Infant Feeding |
| --- |
| 2. (Problems OR Barriers OR Difficulties OR Determinants) OR Early Discontinuation OR Early Cessation OR Early Termination (Facilitate* OR Promote* OR Support OR Motivate*) OR Duration OR Optimal OR Maintenance OR Guideline Adherence). |
| 3. (Ethiopia * OR all regions*OR city administrations (All Fields) OR 9 regions*or two city administrations (All Fields) OR Ethiopia) OR Amhara OR Tigray OR Oromia OR Somali OR Afar OR Benishangul OR Gambelia OR Harari OR SNNP and Addis-Ababa OR Dir Diwa) |
| 4. (English) |
| 5. (Article OR Dissertation OR grey literatures) |
| 6. (#1 OR #2 OR #3 OR #4 OR #5) AND #6)) |
