# Supplementary material for: Early cessation of exclusive breastfeeding and associated factors in Ethiopia: a systematic review and meta-analysis
Source: Front Nutr. 2025 Apr 25;12:1500077. doi: 10.3389/fnut.2025.1500077 (PMC12089787; doi:10.3389/fnut.2025.1500077)
Supplement: Supplementary file 2 [file Table_2.docx]

| sn | Authors | Selection Domain (Max: 4 | Comparability Domain (Max: 2) | Outcome/Exposure Domain (Max: 3) | Total NOS Score (Max: 9) | Risk of Bias Level | Comments on Key Bias Indicators |
| --- | --- | --- | --- | --- | --- | --- | --- |
| 1 | Beyene et al 2019  (18) | 3 | 2 | 3 | 8 | Low Risk | non-responders were not adequately characterized. |
| 2 | Kelkay B etal 2020. (19) | 3 | 2 | 2 | 7 | Low Risk | leaving room for residual bias. |
| 3 | Getachew D et al. 2023 (34) | 4 | 1 | 3 | 8 | Low Risk | leaving room for residual bias. |
| 4 | Adugnaw E et al. 2023  (17) | 3 | 1 | 3 | 7 | Low Risk | leaving room for residual bias  -unaddressed non-response bias |
| 5 | Ibrahim KH etal. 2023  (21) | 4 | 2 | 3 | 9 | Low Risk | Non |
| 6 | Temesgen K etal.2023.  (16) | 3 | 2 | 3 | 8 | Low Risk | unaddressed non-response bias |
| 7 | Yeneabat T etal. 2014 (24) | 4 | 2 | 3 | 9 | Low Risk | Non |
| 8 | Kebede T et al. 2020 (20) | 4 | 2 | 3 | 9 | Low Risk | Non |
| 9 | Techane KT etal. 2022  (33) | 4 | 2 | 3 | 9 | Low Risk | Non |

**Table S2:** Quality Assessment of Included Studies Using the Newcastle-Ottawa Scale (NOS)
